# LncRNA MIR4435-2HG predicts poor prognosis in patients with colorectal cancer

**DOI:** 10.7717/peerj.6683

**Published:** 2019-04-02

**Authors:** Wen Ouyang, Linlin Ren, Guohong Liu, Xiaosa Chi, Hongyun Wei

**Affiliations:** 1 The Second Clinical Medical College, Zhujiang Hospital, Southern Medical University, Guangzhou, Guangdong, China; 2 Department of Gastroenterology, Affiliated hospital of Qingdao University, Qingdao, Shandong, China

**Keywords:** LncRNA MIR4435-2HG, Prognosis, Colorectal cancer

## Abstract

**Background:**

LncRNA MIR4435-2HG is observed in a variety of cancers, while its role in colorectal cancer is unknown. We aimed to demonstrate the relationship between MIR4435-2HG and colorectal cancer based on The Cancer Genome Atlas (TCGA) database.

**Materials and Methods:**

Patients with colorectal cancer were collected from TCGA. We compared the expression of MIR4435-2HG in colorectal cancer and normal tissues with Wilcoxon rank sum test, and logistic regression was used to evaluate the relationship between MIR4435-2HG and clinicopathological characters. Moreover, Kaplan–Meier and Cox regression was performed to evaluate the correlation between MIR4435-2HG and survival rate. Gene set enrichment analysis (GSEA) was also conducted to annotate biological function of MIR4435-2HG.

**Results:**

MIR4435-2HG level was elevated in colorectal cancer tissues. Increased level of MIR4435-2HG was significantly correlated with TNM stage (OR = 1.66 for T1/T2 vs. T3/T4; OR = 1.68 for N0 vs. N1/N2), stage (OR = 1.66 for stage 1/2 vs. stage 3/4), and carcinoembryonic antigen level before treatment (OR = 1.70 for <5 vs. ≥5) (all *P*-value <0.05). High MIR4435-2HG expression had a poorer progression-free survival (*p* = 0.048), and overall survival (OS) (*P* = 0.028), which were validated in the GSE92921 and GSE29621 datasets. MIR4435-2HG expression (*P* = 0.040, HR = 1.955 (95% CI [1.031–3.710])) was independently correlated with OS. GSEA demonstrated that the P38/MAPK pathway, the VEGF pathway, the cell adhesion molecules cams, the NOD-like receptor signaling pathway, the cell surface interactions at the vascular wall, and integrin cell surface interactions were differentially enriched in MIR4435-2HG high expression phenotype.

**Conclusions:**

Increased MIR4435-2HG might be a potential biomarker for the diagnosis and prognosis of colorectal cancer. Moreover, MIR4435-2HG might participate in the development of colorectal cancer via the P38/MAPK and VEGF pathway.

## Introduction

The prevalence of colorectal cancer is increasing worldwide (Altobelli, D’Aloisio & Angeletti, 2016). Surgery is regarded as the standard of care for locally colorectal cancer, while many patients experience recurrence after surgery. For patients with distant metastasis, chemotherapy and radiotherapy are recommended. However, the response to these treatments are variable and unpredictable for individual patients. In addition, the cost for colorectal cancer treatments becomes a heavy burden of both the patients and the society. The prognosis is mainly dependent on tumor stage, patients with early tumors had higher 5-year survival rates, therefore, it is necessary to identify reliable predictors that are related to tumor stage and prognosis, and provide new targets for treatments, diagnosis, and prognostic evaluation. Although various biomarkers have been considered to be related with colorectal cancer in clinical practice (Gires, 2011), such as carcinoembryonic antigen (CEA) and carbohydrate antigen 199 (CA199) (Chen et al., 2013), their reliability remains controversial.

MIR4435-2HG, also known as AGD2, MORRBID, LINC00978, MIR4435-1HG, lncRNA-AWPPH, has been regarded as a new oncogenic lncRNA in many types of cancer, like breast cancer and bladder cancer (Zhu et al., 2018). A recent study showed higher expressed MIR4435-2HG was observed in triple-negative breast cancer compared to paired adjacent healthy tissues, and contributed to cancer cell proliferation (Wang et al., 2018). In glioblastoma, MIR4435-2HG, as a homolog of LINC00152, was found to be related with aggressive tumor characters via promoting cellular invasion and predict poor patient survival (Reon et al., 2018). Additionally, the expression of lncRNA MIR4435-2HG was positively related to tumor grade and lymph node metastasis in lung cancer, and promoted lung cancer cell invasion and proliferation as well (Qian et al., 2018). Besides, lncRNA MIR4435-2HG was identified in plasma samples of gastric cancer patients, and be regarded as a diagnostic and prognostic biomarker in patients with gastric carcinoma (Ke et al., 2017). However, few literature about the relationship between MIR4435-2HG and colorectal cancer has been reported so far.

Therefore, we aimed to demonstrate the correlation between MIR4435-2HG and colorectal cancer, and analyze the prognostic role of MIR4435-2HG in colorectal cancer based on The Cancer Genome Atlas (TCGA). To achieve this goal, we analyzed the expression level of LncRNA MIR4435-2HG in colorectal cancer and normal tissues based on TCGA. And the correlation between LncRNA MIR4435-2HG and prognostic values was performed. Furthermore, MIR4435-2HG related biological pathways involved in colorectal cancer were detected using Gene set enrichment analysis (GSEA).

The present study showed increased MIR4435-2HG was related to poor prognosis of colorectal cancer, and P38/MAPK pathway, VEGF pathway, cell adhesion molecules cams, Nod like receptor signaling pathway, cell surface interactions at the vascular wall, and integrin cell surface interactions were associated with LncRNA MIR4435-2HG expression phenotype using GSEA.

## Materials and Methods

### RNA sequencing, micro array, and clinic information from TCGA and GEO data repository

In the TCGA database, 459 patients with colonic adenocarcinoma and 172 cases with rectal adenocarcinoma were collected. Finally, 580 cases were included in the further analysis, while cases without RNA-seq data (*n* = 9) and with overall survival (OS) less than 30 days (*n* = 42) were excluded. The related RNA-seq data and clinicopathological data were extracted. The expression of lncRNA MIR4435-2HG in colorectal adenocarcinoma were analyzed and compared with that in adjacent normal tissues. Considering the fact that the relationship between LncRNA MIR4435-2HG expression and the development of a tumor is independent of the follow-up days, we used 622 RNA-seq data for further association analysis, and 580 data for survival analysis. The characteristics of patients including gender, race, differentiation, venous or perineural invasion, preoperative pretreatment, TNM stage, and tumor location were recorded. Some of them were not available, which were treated as missing value. In addition, the Gene Expression Omnibus (GEO) database was used to validate the association between MIR4435-2HG expression and colorectal cancer outcome. Two raw gene expression datasets [GSE92921, GSE29621] were downloaded from GEO for further study.

### Gene set enrichment analysis

In this study, GSEA was used to elucidate the significant survival difference between high- and low- MIR4435-2HG groups. The number of gene set permutations were 1,000 times for each analysis. The expression level of lncRNA MIR4435-2HG was used as a phenotype label. The pathways enrichment was analyzed based on nominal *P* value and normalized enrichment score (NES).

### Statistical analysis

Statistical analysis was performed using R (v.3.5.1) (R Core Team, 2018). Comparison of the expression of lncRNA MIR4435-2HG between colorectal adenocarcinoma and normal groups was performed using Wilcoxon rank sum tests, and adjacent groups with Wilcoxon signed-rank tests. We divided subjects into two groups with gene expression above median value vs. subjects with gene expression below median value. The relationship between clinical pathologic features and lncRNA MIR4435-2HG was analyzed with the Wilcoxon rank sum test or Kruskal–Wallis test and logistic regression. Clinicopathologic characteristics associated with OS in lncRNA MIR4435-2HG patients using Cox regression and the Kaplan–Meier method. Multivariate Cox analysis was used to compare the influence of lncRNA MIR4435-2HG expression on survival along with other clinical characteristics.

## Results

### Clinical characteristics

The characteristics of patients including gender, race, differentiation, venous or perineural invasion, preoperative pretreatment, TNM stage, and tumor location were collected, as shown in Table 1. A total of 264 female patients and 316 male patients were analyzed in the present study, including 281 white patients and 74 non-white patients. The cancer status included 247 tumor free (48.34%) and 264 with tumor (51.66%). Before treatment, patients with CEA level less than five took a percentage of 64% (*n* = 240), and 156 cases (31.52%) had history of colon polyps. Stage I disease was found in 102 patients (18.21%), stage II in 206 (36.79%), stage III in 168 (30.00%), and stage IV in 84 (15.00%). Most tumors (72.93%, *n* = 423) were of colonic adenocarcinoma, and 27.07% (*n* = 157) were rectal adenocarcinoma. The topography distribution included 3.29% T1 (*n* = 19), 17.82% T2 (*n* = 103), 68.17% T3 (*n* = 394), and 10.73% T4 (*n* = 62). A total of 123 cases (24.50%) had venous invasion, and 57 (26.03%) perineural invasion. A total of 83 cases (16.12%) had distant metastases.

**Table 1:** Characteristics of patients with colorectal adenocarcinoma based on TCGA.

Characteristics		Number of cases	Percentages (%)
Topography (T)	T1	19	3.29
	T2	103	17.82
	T3	394	68.17
	T4	62	10.73
Lymph node (N)	N0	327	56.67
	N1	144	24.96
	N2	106	18.37
Metastasis (M)	M0	432	83.88
	M1	83	16.12
Stage	Stage 1	102	18.21
	Stage 2	206	36.79
	Stage 3	168	30.00
	Stage 4	84	15.00
CEA level before treatment	<5	240	64.00
	=>5	135	36.00
Colon polyps at diagnosis	No	199	69.58
	Yes	87	30.42
History of colon polyps	No	339	68.48
	Yes	156	31.52
Histological type	Colon cancer	423	72.93
	Rectal cancer	157	27.07
Perineural invasion	No	162	73.97
	Yes	57	26.03
Status	Tumor free	247	48.34
	With tumor	264	51.66
Venous invasion	No	379	75.50
	Yes	123	24.50
Race	Not_white	74	20.85
	White	281	79.15
Gender	Female	264	45.52
	Male	316	54.48

### LncRNA MIR4435-2HG were highly expressed in colorectal tissues

**Figure 1:**
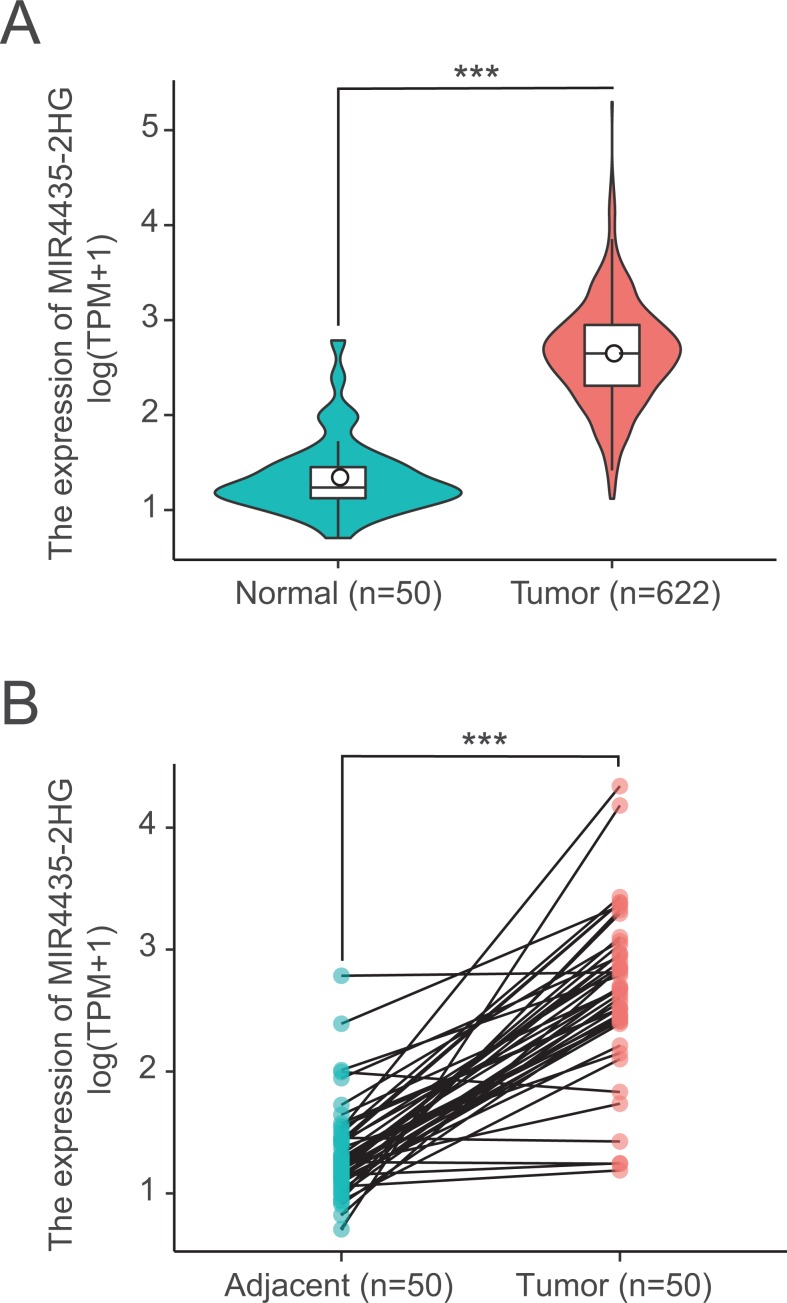
LncRNA MIR4435-2HG is significantly up-regulated in colorectal cancer than normal or adjacent normal tissues. (A) MIR4435-2HG showed significantly higher expression in cancer tissues than in normal tissues (*P* < 0.001). (B) showed MIR4435-2HG was prominently overexpressed in colorectal cancer (*P* < 0.001) compared with 50 pairs non-cancerous adjacent tissues using Wilcoxon singed-rank test.

We next examined LncRNA MIR4435-2HG expression in 622 colorectal cancer tissues and 50 normal tissues using Wilcoxon rank sum test. MIR4435-2HG showed significantly higher expression in cancer tissues than in normal tissues (*P* < 0.001) (Fig. 1A). In addition, we further analyzed the expression of LncRNA MIR4435-2HG in 50 pairs colorectal cancer tissues and non-cancerous adjacent tissues using Wilcoxon singed-rank test, the result showed MIR4435-2HG was prominently overexpressed in colorectal cancer (*P* < 0.001) (Fig. 1B), indicating LncRNA MIR4435-2HG may facilitate colorectal carcinogenesis.

### Correlations between LncRNA MIR4435-2HG expression and clinical characteristics in colorectal cancer patients

**Figure 2:**
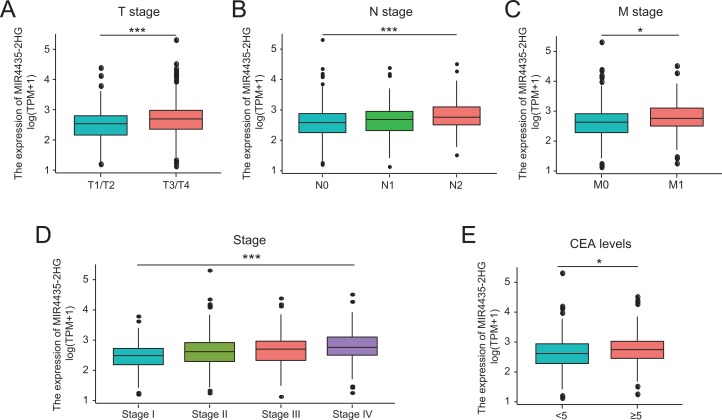
Association with LncRNA MIR4435-2HG expression and clinicopathologic characteristics. (A) T stage, (B) N stage, (C) M stage, (D) Clinical stage, (E) CEA levels before treatment. T, topography distribution; N, lymph node metastasis; M, distant metastasis.

A total of 580 colorectal cancer samples with LncRNA MIR4435-2HG expression data were analyzed from TCGA. Increased expression of MIR4435-2HG was correlated significantly with the grade of topography distribution (*P* < 0.001), lymph node metastasis (*P* < 0.001), stage (*P* < 0.001), and CEA level before treatment (*P* = 0.013), as shown in Figs. 2A–2E.

Increased LncRNA MIR4435-2HG expression in colorectal cancer was significantly associated with TNM stage (OR = 1.66 for T1/T2 vs. T3/T4, *P* = 0.015; OR = 1.68 for N0 vs. N1/N2, *P* = 0.002), stage (OR = 1.66 for stage1/2 vs. stage3/4, *P* = 0.003), and CEA level before treatment (OR = 1.70 for <5 vs. ≥5, *P* = 0.015) (Table 2). Univariate analysis revealed that increased LncRNA MIR4435-2HG expression (based on median value) was associated with poor prognostic clinicopathologic characteristics using logistic regression (Table 2). These results indicated colorectal cancer with increased LncRNA MIR4435-2HG expression is prone to progress to a more advanced stage and lymph node metastasis.

**Table 2:** LncRNA MIR4435-2HG expression associated with clinical pathological characteristics (logistic regression).

Characteristics	Total (*N*)	Odds ratio in MIR4435-2HG expression	*P*-value
T (T1/T2 vs. T3/T4)	578	1.66 (1.11–2.49)	0.015
N (N0 vs. N1N2)	577	1.68 (1.2–2.34)	0.002
M (M0 vs. M1)	515	1.54 (0.96–2.49)	0.077
Stage (Stage 1/2 vs. Stage 3/4)	560	1.66 (1.19–2.32)	0.003
CEA level before treatment (<5 vs. =>5)	375	1.7 (1.11–2.6)	0.015
Colon polyps (No vs. Yes)	286	1.08 (0.65–1.8)	0.766
History of colon polyps (No vs. Yes)	495	1.19 (0.82–1.75)	0.360
Histological type (Colon cancer vs. Rectal cancer)	580	0.82 (0.57–1.19)	0.304
Perineural invasion (No vs. Yes)	219	1.48 (0.81–2.74)	0.207
Status (Tumor free vs. With tumor)	511	0.99 (0.7–1.4)	0.949
Venous invasion (No vs. Yes)	502	1.38 (0.92–2.09)	0.120

### Role of LncRNA MIR4435-2HG in colorectal cancer patient survival

**Figure 3:**
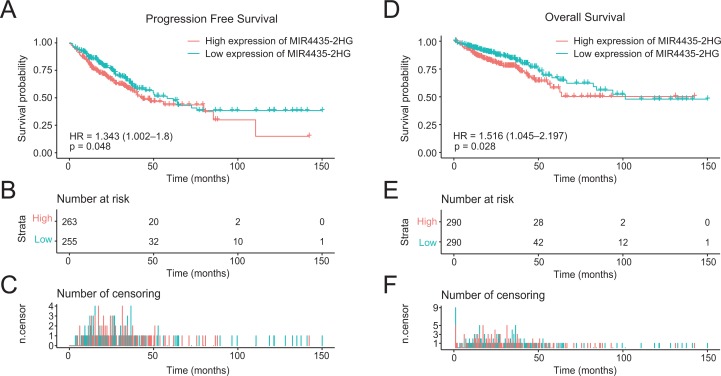
High expression of LncRNA MIR4435-2HG is associated with poor PFS and OS in patients with colorectal cancer. (A) The Kaplan–Meier curves, (B) number at risk, and (C) number of censoring of PFS in colorectal cancer. (D) The Kaplan–Meier curves, (E) number at risk, and (F) number of censoring of OS in colorectal cancer. PFS, progression free survival; OS, over survival.

In the 580 colorectal cancer patients included in the study, the median follow-up time is 22.7 months. Progression-free survival (PFS) was significantly poorer in patients with high LncRNA MIR4435-2HG expression than those with low LncRNA MIR4435-2HG expression (*P* = 0.048) (Figs. 3A–3C), similar result is observed in OS analysis (*P* = 0.028) (Figs. 3D–3F). In addition, patients with high LncRNA MIR4435-2HG expression owned lower median PFS (43.5 months) compared with low expression group (58.0 months), while no median OS was found in high LncRNA MIR4435-2HG expression group. To validate the association between MIR4435-2HG expression and colorectal cancer outcome, we also used the GSE92921 and GSE29621 datasets from the GEO database to verify it. The result demonstrated that disease-free survival (DFS) was significantly poorer in patients with high LncRNA MIR4435-2HG expression than those with low LncRNA MIR4435-2HG expression (*P* = 0.044) based on GSE92921 (Figs. S1A–S1C), and a similar result is observed in OS analysis (*P* = 0.021) based on GSE29621 (Figs. S1D–S1F). We next performed univariate analysis of prognostic factors for OS with the Cox regression model (Table 3). High LncRNA MIR4435-2HG expression levels were associated with worse OS (*P* = 0.028, hazard ratio [HR] = 1.516 (95% CI [1.045–2.197])), older patients (*P* = 0.004, HR = 1.820 (95% CI [1.215–2.726])), higher CEA level (*P* < 0.001, HR = 2.563 (95% CI [1.542–4.262])), higher TNM stage (T: *P* = 0.004, HR = 2.716 (95% CI [1.372–5.377]); N: *P* < 0.001, HR = 2.991 (95% CI [2.027–4.414]); M: *P* < 0.001, HR = 4.737 (95% CI [3.156–7.11])), higher disease stage (*P* < 0.001, HR = 3.541 (95% CI [2.331–5.377])), and venous invasion (*P* < 0.001, HR = 2.492 (95% CI [1.666–3.728])) (Table 3).

**Table 3:** Univariate regression and multivariate survival model of prognostic covariates in patients with colorectal cancer.

Characteristics	Harzard ratio	CI95	*P*-value
Univariate analysis			
T (T1/T2 vs. T3/T4)	2.716	1.372–5.377	0.004
N (N0 vs. N1N2)	2.991	2.027–4.414	<0.001
M (M0 vs. M1)	4.737	3.156–7.11	<0.001
Stage (Stage 1/2 vs. Stage 3/4)	3.541	2.331–5.377	<0.001
CEA level before treatment (<5 vs. =>5)	2.563	1.542–4.262	<0.001
Colon polyps (No vs. Yes)	1.278	0.738–2.214	0.381
History of colon polyps (No vs. Yes)	0.751	0.453–1.244	0.266
Histological type (Colon cancer vs. Rectal cancer)	0.851	0.549–1.318	0.469
Perineural invasion (No vs. Yes)	1.609	0.826–3.134	0.162
Status (Tumor free vs. With tumor)	2.704	1.698–4.306	<0.001
Venous invasion (No vs. Yes)	2.492	1.666–3.728	<0.001
Race (Not_White vs. White)	0.911	0.518–1.599	0.745
Gender (Female vs. Male)	1.089	0.754–1.573	0.648
Age (<65 vs. >=65)	1.820	1.215–2.726	0.004
Height (<170 vs. >=170)	0.772	0.464–1.285	0.319
Weight (<80 vs. >=80)	0.704	0.426–1.163	0.171
MIR4435-2HG (low vs. high)	1.516	1.045–2.197	0.028
Multivariate analysis			
T (T1/T2 vs. T3/T4)	1.251	0.357–4.383	0.726
N (N0 vs. N1N2)	0.198	0.055–0.72	0.014
M (M0 vs. M1)	1.317	0.562–3.085	0.526
Stage (Stage 1/2 vs. Stage 3/4)	17.891	3.407–93.956	0.001
CEA level before treatment (<5 vs. =>5)	1.53	0.81–2.89	0.19
Status (Tumor free vs. With tumor)	3.776	1.705–8.361	0.001
Venous invasion (No vs. Yes)	2.477	1.315–4.666	0.005
Age (<65 vs. >=65)	3.129	1.535–6.38	0.002
MIR4435-2HG (Low vs. High)	1.955	1.031–3.71	0.04

We then performed multivariate analysis with Cox regression model (Table 3). LncRNA MIR4435-2HG expression (*P* = 0.040, HR = 1.955 (95% CI [1.031–3.710])), age (*P* = 0.002, HR = 3.129 (95% CI [1.535–6.380])), status (*P* = 0.001, HR = 3.776 (95% CI [1.705–8.361])), lymph node metastasis (*P* = 0.014, HR = 0.198 (95% CI [0.055–0.72])), and disease stage (*P* = 0.001, HR = 17.891 (95% CI [3.407–93.956])) were independently correlated with OS in multivariate analysis (Table 3). The above data indicated LncRNA MIR4435-2HG is a prognostic factor and increased LncRNA MIR4435-2HG level is associated with poor OS.

### MIR4435-2HG-related signaling pathways based on GSEA

Gene set enrichment analysis was used to identify signaling pathways involved in colorectal cancer between low and high LncRNA MIR4435-2HG expression data sets, and demonstrated significant differences (FDR < 0.05, NOM *P*-value < 0.05) in enrichment of MSigDB Collection (c2.cp.v6.2.symbols.gmt).

**Figure 4:**
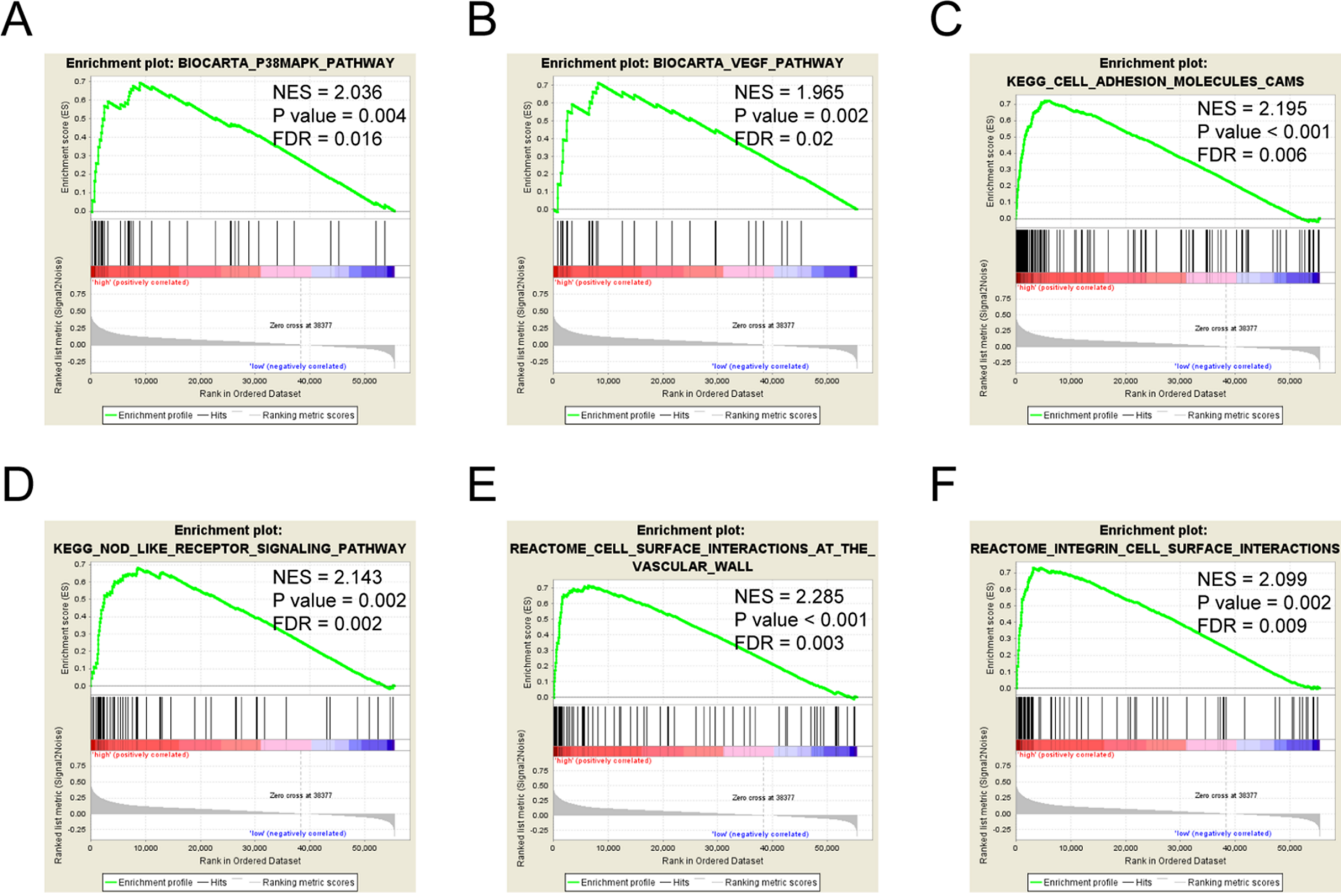
Enrichment plots from gene set enrichment analysis (GSEA). GSEA results showing P38/MAPK pathway (A), VEGF pathway (B), cell adhesion molecules cams (C), NOD-like receptor signaling pathway (D), cell surface interactions at the vascular wall (E), integrin cell surface interactions (F) differentially enriched in MIR4435-2HG-related colorectal cancer. NES, normalized ES; FDR, false discovery rate.

Six pathways, including the P38/MAPK pathway, the VEGF pathway, the cell adhesion molecules cams, the NOD like receptor signaling pathway, the cell surface interactions at the vascular wall, and integrin cell surface interactions showed significantly differential enrichment in LncRNA MIR4435-2HG high expression phenotype based on NES, NOM *P* value, and FDR value (Figs. 4A–4F; Table 4), indicating the potential role of LncRNA MIR4435-2HG in the development of colorectal cancer.

**Table 4:** Gene sets enriched in phenotype high.

MSigDB collection	Gene set name	NES	NOM *P*-val	FDR *Q*-val
c2.cp.reactome/biocarta/kegg.v6.2.symbols.gmt	BIOCARTA_P38/MAPK_PATHWAY	2.036	0.004	0.016
	BIOCARTA_VEGF_PATHWAY	1.965	0.002	0.02
	KEGG_CELL_ADHESION_MOLECULES_CAMS	2.195	<0.001	0.006
	KEGG_NOD_LIKE_RECEPTOR_SIGNALING_PATHWAY	2.143	0.002	0.002
	REACTOME_CELL_SURFACE_INTERACTIONS_AT_THE_VASCULAR_WALL	2.285	<0.001	0.003
	REACTOME_INTEGRIN_CELL_SURFACE_INTERACTIONS	2.099	0.002	0.009

**Note:**

NES, normalized ES; NOM *P*-val, normalized *P*-value; FDR, false discovery rate.

## Discussion

Overwhelming studies recently have shown that LncRNA MIR4435-2HG plays a key role in various cancer types, can promote cancer cell proliferation and invasion, and might act as an oncogenic gene. LncRNA MIR4435-2HG contributes to esophageal cancer cell growth via regulating the expression of P53, which also participates in the development of colorectal cancer (Zhang et al., 2018). However, little literature has explored the correlation between LncRNA MIR4435-2HG and colorectal cancer. Therefore, the present study aimed to clarify the expression of LncRNA MIR4435-2HG in colorectal cancer tissues, and its potential therapeutic and prognostic value.

In this study, we collected colorectal cancer data based on high throughput RNA sequencing from TCGA database, and demonstrated LncRNA MIR4435-2HG was significantly up-regulated in colorectal cancer tissues compared with normal or adjacent normal tissues. Moreover, increased LncRNA MIR4435-2HG in colorectal cancer tissues was positively correlated with high clinical stage, advanced TNM stage, and poor prognosis. Furthermore, we investigated the function of LncRNA MIR4435-2HG in colorectal cancer tissues using GSEA, and the result showed in LncRNA MIR4435-2HG high expression phenotype, pathways, like the P38/MAPK pathway, the VEGF pathway, the cell adhesion molecules cams, NOD-like receptor signaling pathway, the cell surface interactions at the vascular wall, and integrin cell surface interactions were found significantly differential enrichment. These pathways were reported to be contributors to cancer cell proliferation, invasion, and metastasis (Dey et al., 2016; Hu et al., 2018; Marsico et al., 2018; Ono & Uede, 2018; Peng et al., 2018), indicating the potential role of LncRNA MIR4435-2HG as new therapeutic and prognostic target in colorectal cancer.

MIR4435-2HG, as a lncRNA, has been reported to promote lung cancer cell proliferation and growth, and MIR4435-2HG knockdown could lead to a cell-cycle arrest and increase the percentage of cells in G0/G1 phage (Yang et al., 2015). Similar result was found in experiment of HeLa cells, which showed LINC00152, a paralog of MIR4435-2HG, contributed to cell division through regulating cell-cycle progression (Nötzold et al., 2017). Furthermore, increased MIR4435-2HG expression level was observed in bladder cancer, and could promote cell proliferation and migration by inhibiting SMAD4 (Zhu et al., 2018). Similarly, the present study demonstrated MIR4435-2HG was up-regulated in colorectal cancer tissues, and correlated with TNM stage, clinic stage, and lymph node metastasis, highlighting the potential role of MIR4435-2HG in the development of colorectal cancer.

In addition, high MIR4435-2HG expression was also reported to have poor disease-free survival rate in breast cancer (Deng et al., 2016). In gastric cancer, MIR4435-2HG was also identified as a biomarker significantly associated with OS (Miao et al., 2017). Analysis of prognosis was also performed in the present study, and high level of MIR4435-2HG indicated poor survival rate as the previous study showed.

Given that limit data about MIR4435-2HG function is shown, we performed functional annotation based on GSEA, and demonstrated that MIR4435-2HG was involved in the P38/MAPK pathway, the VEGF pathway, cell adhesion molecules cams, NOD-like receptor signaling pathway, the cell surface interactions at the vascular wall, and integrin cell surface interactions. Recent researches showed that strong phosphorylated P38/MAPK in colorectal cancer was an independent factor associated with poorer survival (Fan et al., 2014; Roseweir et al., 2018). Angiogenesis is a necessary step in tumor metastasis, and VEGF is a well-known angiogenesis factor (Mar et al., 2015). Inhibiting VEGF pathway could lead to reduced colorectal cancer angiogenesis, and decreased colorectal cancer proliferation, migration (Xu et al., 2018), indicating that MIR4435-2HG might promote colorectal cancer cell growth, metastasis, and poor survival via P38/MAPK and VEGF pathway.

Although our approach in the current study improved our understanding the relationship between MIR4435-2HG and colorectal cancer, there were still some limitations. First, to clarify the specific role of MIR4435-2HG in the development of colorectal cancer comprehensively, all kinds of clinical factors should be considered, such as the details on treatments received by patients involved. However, this kind of information lacks or inconsistency treatments exist in public databases because the experiments were performed in different laboratories. Second, the number of healthy subjects used as controls is largely different from that of cancer patients in the current study, so additional studies are required to keep sample size balance. All in all, although multi-center study in public databases intends to complement the short-comings of single center study, retrospective studies still have their own limits, especially non-uniform intervening measures, and lacking of some information. Therefore, prospective study should be performed in the future study to avoid analysis bias led by the retrospective nature of the current study.

The current study was performed based on RNA sequencing from TCGA database, therefore, we cannot illustrate the expression of MIR4435-2HG from protein level, and also cannot clearly evaluate the direct mechanisms of MIR4435-2HG involved in the development of colorectal cancer. So further studies about direct mechanisms in colorectal cancer are needed.

## Conclusions

We observed increased MIR4435-2HG in colorectal cancer, which was also related to poor OS. Moreover, MIR4435-2HG might participate in the development of colorectal cancer via the P38/MAPK and VEGF pathway. The current study partially unveiled the roles of MIR4435-2HG in colorectal cancer and provided a potential biomarker for the diagnosis and prognosis of colorectal cancer.

## Supplemental Information

10.7717/peerj.6683/supp-1 Supplemental Information 1 raw data.

10.7717/peerj.6683/supp-2 Supplemental Information 2 High expression of LncRNA MIR4435-2HG is associated with poor DFS and OS in patients with colorectal cancer based on GEO database. (A) The Kaplan–Meier curve, (B) Number at risk, and (C) Number of censoring of DFS in colorectal cancer based on GSE92921 dataset. (D) The Kaplan–Meier curve, (E) Number at risk, and (F) Number of censoring OS in colorectal cancer based on GSE29621 dataset. DFS: disease-free survival; OS: over survival.

10.7717/peerj.6683/supp-3 Supplemental Information 3 GSE29621 raw data extracted from GEO applied for Fig. S1.

10.7717/peerj.6683/supp-4 Supplemental Information 4 GSE92921 raw data extracted from GEO applied for Fig. S1.

10.7717/peerj.6683/supp-5 Supplemental Information 5 TCGA raw data extracted from TCGA applied for data analyses.

## Additional Information and Declarations

### Data Availability

The following information was supplied regarding data availability: The raw measurements are available in File S1.
