# Supplementary material for: LncRNA MIR4435-2HG predicts poor prognosis in patients with colorectal cancer
Source: PeerJ. 2019 Apr 2;7:e6683. doi: 10.7717/peerj.6683 (PMC6450379; doi:10.7717/peerj.6683)
Supplement: Supplemental Information 2 — (A) The Kaplan–Meier curve, (B) Number at risk, and (C) Number of censoring of DFS in colorectal cancer based on GSE92921 dataset. (D) The Kaplan–Meier curve, (E) Number at risk, and (F) Number of censoring OS in colorectal cancer based on GSE29621 dataset. DFS: disease-free survival; OS: over survival. [file peerj-07-6683-s002.pdf]

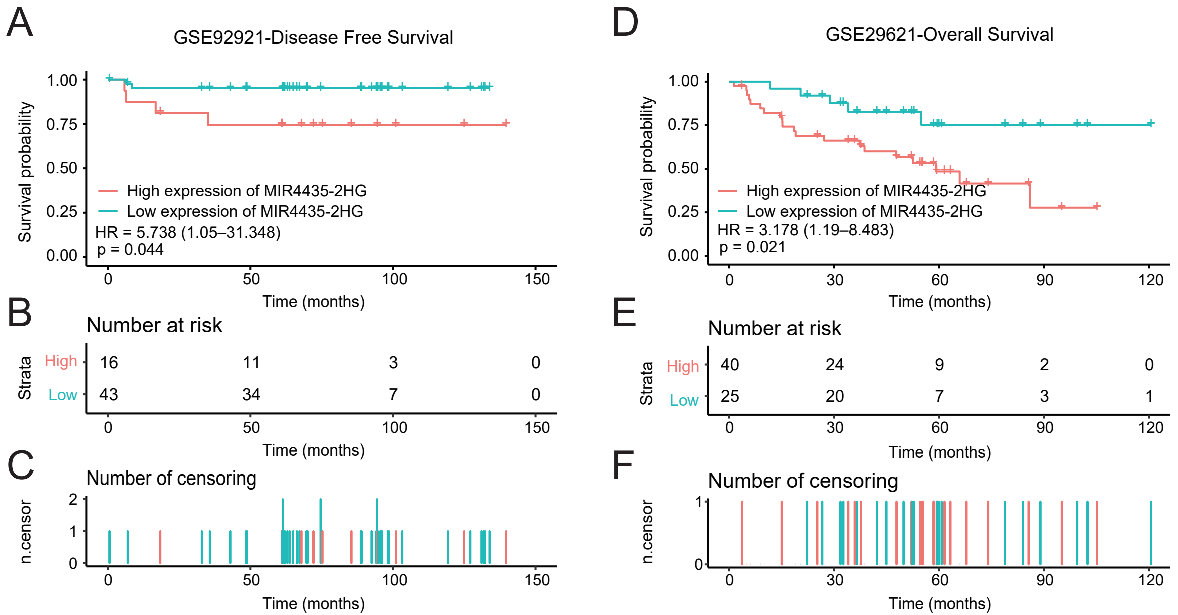
A
GSE92921-Disease Free Survival
Survival probability
1.00
0.75
0.50
0.25
0.00
High expression of MIR4435-2HG
Low expression of MIR4435-2HG
HR = 5.738 (1.05–31.348)
p = 0.044
0
50
100
150
Time (months)
B
Number at risk
Strata
High
Low
16
11
3
0
43
34
7
0
Time (months)
C
Number of censoring
n.censor
Time (months)
D
GSE29621-Overall Survival
Survival probability
High expression of MIR4435-2HG
Low expression of MIR4435-2HG
HR = 3.178 (1.19–8.483)
p = 0.021
0
30
60
90
120
Time (months)
E
Number at risk
Strata
High
Low
40
24
9
2
0
25
20
7
3
1
Time (months)
F
Number of censoring
n.censor
Time (months)
